# Diethyl 2,6-dimethyl-4-[4-(3-phenyl­acrylo­yloxy)phen­yl]-1,4-dihydro­pyridine-3,5-dicarboxyl­ate hemihydrate

**DOI:** 10.1107/S1600536813004108

**Published:** 2013-02-16

**Authors:** P. Sharmila, C. Suresh Kumar, Karthik Ananth, S. Narasimhan, S. Aravindhan

**Affiliations:** a Department of Physics, Presidency College, Chennai 600 005, India; b Asthagiri Herbal Research Foundation, Perungudi, Chennai 600 096, India

## Abstract

In the title ester derivative, C_28_H_29_NO_6_·0.5H_2_O, the 1,4-dihydro­pyridine ring has a flattened boat conformation. The mean plane is almost perpendicular to the attached benzene ring, making a dihedral angle of 86.87 (9)°. The terminal phenyl ring is inclined to the central benzene ring by 67.56 (12)°. In the crystal, mol­ecules are bridged *via* O—H⋯O hydrogen bonds involving the partially occupied water mol­ecule, and this arrangement is strengthened by a pair of N—H⋯O hydrogen bonds and C—H⋯O inter­actions. The ethyl atoms of one of the ethyl ester groups are disordered over two sites with an occupancy ratio of 0.716 (5):0.284 (5).

## Related literature

For the biological activity of ester derivatives, see: Bi *et al.* (2012 ▶); Bartzatt *et al.* (2004 ▶); Anadu *et al.* (2006 ▶).
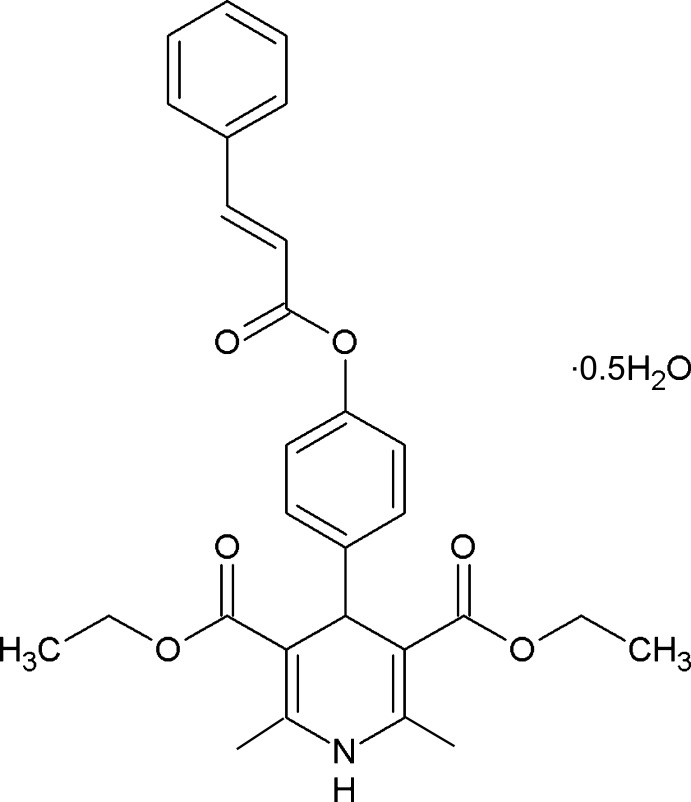


## Experimental

### 

#### Crystal data

C_28_H_29_NO_6_·0.5H_2_O
*M* *_r_* = 484.53
Monoclinic, 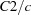

*a* = 25.4905 (11) Å
*b* = 8.6166 (4) Å
*c* = 23.2902 (10) Å
β = 92.235 (2)°
*V* = 5111.6 (4) Å^3^
*Z* = 8
Mo *K*α radiation
μ = 0.09 mm^−1^
*T* = 293 K
0.25 × 0.20 × 0.20 mm

#### Data collection

Bruker Kappa APEXII CCD diffractometer
Absorption correction: multi-scan (*SADABS*; Bruker 2004 ▶) *T*_min_ = 0.979, *T*_max_ = 0.983
24059 measured reflections
5036 independent reflections
3271 reflections with *I* > 2σ(*I*)
*R*_int_ = 0.027

#### Refinement

*R*[*F* ^2^ > 2σ(*F* ^2^)] = 0.048
*wR*(*F* ^2^) = 0.146
*S* = 1.09
5036 reflections
344 parameters
2 restraints
H atoms treated by a mixture of independent and constrained refinement
Δρ_max_ = 0.18 e Å^−3^
Δρ_min_ = −0.31 e Å^−3^

### 

Data collection: *APEX2* (Bruker, 2004 ▶); cell refinement: *APEX2* and *SAINT* (Bruker, 2004 ▶); data reduction: *SAINT* and *XPREP* (Bruker, 2004 ▶); program(s) used to solve structure: *SHELXS97* (Sheldrick, 2008 ▶); program(s) used to refine structure: *SHELXL97* (Sheldrick, 2008 ▶); molecular graphics: *ORTEP-3 for Windows* (Farrugia, 2012 ▶); software used to prepare material for publication: *PLATON* (Spek, 2009 ▶).

## Supplementary Material

Crystal structure: contains datablock(s) I, global. DOI: 10.1107/S1600536813004108/su2546sup1.cif

Structure factors: contains datablock(s) I. DOI: 10.1107/S1600536813004108/su2546Isup2.hkl

Supplementary material file. DOI: 10.1107/S1600536813004108/su2546Isup3.cml

Additional supplementary materials:  crystallographic information; 3D view; checkCIF report

## Figures and Tables

**Table 1:** Hydrogen-bond geometry (Å, °)

*D*—H⋯*A*	*D*—H	H⋯*A*	*D*⋯*A*	*D*—H⋯*A*
N1—H1*N*⋯O1^i^	0.87 (2)	2.31 (2)	3.148 (2)	163 (2)
O1*W*—H1*W*⋯O5	1.04	1.81	2.615 (6)	132
C14—H14⋯O1*W* ^ii^	0.93	2.50	3.140 (6)	126

Symmetry codes: (i) 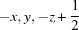; (ii) 
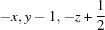
.

**Fig. 1.**
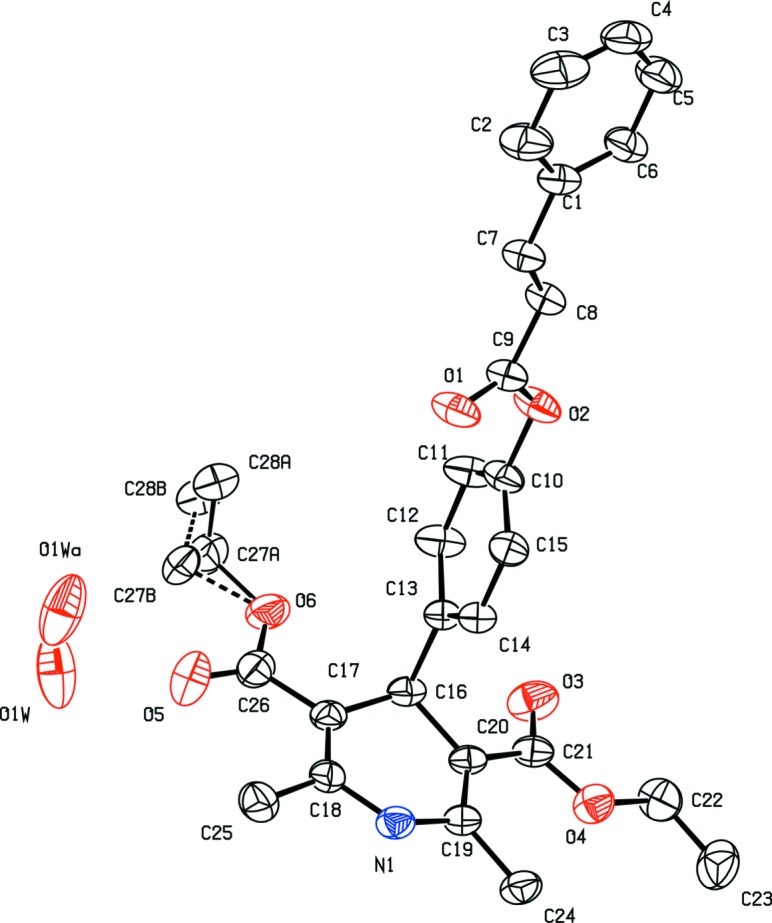
The molecular structure of the title compound, with the atom numbering. Displacement ellipsoids are drawn at the 30% probability level [H atoms have been omitted for clarity].

**Fig. 2.**
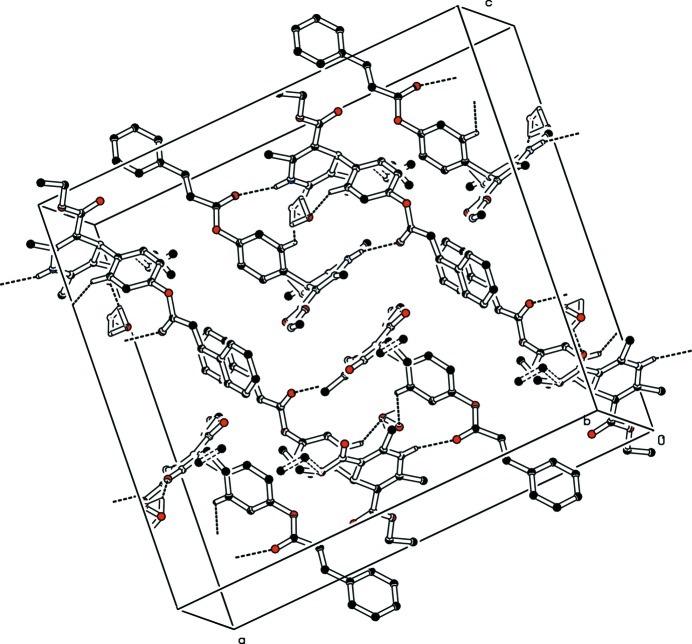
Crystal packing of the title compound viewed along the b axis. Hydrogen bonds are shown as dashed lines [for the sake of clarity, H atoms not involved in these interactions have been omitted].

## supplementary crystallographic information

### Comment

Ester derivatives of many compounds exhibit a variety of pharmacological properties, for example anticancer, antitumor and antimicrobial activities (Anadu *et al.*, 2006; Bi *et al.*, 2012; Bartzatt *et al.*, 2004). In view of their importance, the title compound was synthesized and we report herein on its crystal structure.

In the title molecule (Fig. 1) the 1,4-dihydropyridine ring (N1/C16-C20) mean plane is almost perpendicular to the attached benzene ring (C10-C15), with a dihedral angle of 86.87 (9)°. This central benzene ring makes a dihedral angle of 67.56 (12)° with the terminal phenyl ring (C1-C6).

In the crystal, molecules are linked via O-H···O hydrogen bonds involving the water molecule, and this arrangement is strengthened by a pair of N-H···O hydrogen bonds and C-H···O interactions (Fig. 2 and Table 1).

### Experimental

A mixture of 3-phenyl-acrylic acid 4-formyl-phenyl ester (0.01 mol), ethyl acetoacetate (0.02 mol), and ammonium acetate (0.03 mol) in ethanol (30 ml) was refluxed for 8 h. After the reaction had completed, the mixture was quenched with an excess of water. The solid precipitated which separated was dried and recrystallized from ethanol, giving colourless block-like crystals.

### Refinement

The CH_3_-CH_2_- atoms of one of the ethylester groups (C27 and C28) are disordered over two sites with an occupancy ratio of 0.716 (5):0.284 (5). The NH H atom was located in a difference Fourier map and refined with *U*_iso_(H) = 1.2U_eq_(N). The C-bound H atoms were positioned geometrically and constrained to ride on their parent atom: C–H = 0.93, 0.98, 0.97 and 0.96 Å for aromatic, methine, methylene and methyl H atoms, respectively, with *U*_iso_(H) = 1.5U_eq_(C) for methyl H atoms and = 1.2U_eq_(C) for other H atoms. An isolated half water molecule (O1W) was refined isotropically with an occupancy of 0.5.

### Crystal data

**Table:**

C_28_H_29_NO_6_·0.5H_2_O	*F*(000) = 2056
*M**_r_* = 484.53	*D*_x_ = 1.259 Mg m^−^^3^
Monoclinic, *C*2/*c*	Mo *K*α radiation, λ = 0.71073 Å
Hall symbol: -C 2yc	Cell parameters from 8834 reflections
*a* = 25.4905 (11) Å	θ = 2.1–31.2°
*b* = 8.6166 (4) Å	µ = 0.09 mm^−^^1^
*c* = 23.2902 (10) Å	*T* = 293 K
β = 92.235 (2)°	Block, colourless
*V* = 5111.6 (4) Å^3^	0.25 × 0.20 × 0.20 mm
*Z* = 8	

### Data collection

**Table:**

Bruker Kappa APEXII CCD diffractometer	5036 independent reflections
Radiation source: fine-focus sealed tube	3271 reflections with *I* > 2σ(*I*)
Graphite monochromator	*R*_int_ = 0.027
ω and φ scan	θ_max_ = 26.0°, θ_min_ = 2.3°
Absorption correction: multi-scan (*SADABS*; Bruker 2004)	*h* = −31→21
*T*_min_ = 0.979, *T*_max_ = 0.983	*k* = −10→10
24059 measured reflections	*l* = −28→27

### Refinement

**Table:**

Refinement on *F*^2^	Primary atom site location: structure-invariant direct methods
Least-squares matrix: full	Secondary atom site location: difference Fourier map
*R*[*F*^2^ > 2σ(*F*^2^)] = 0.048	Hydrogen site location: inferred from neighbouring sites
*wR*(*F*^2^) = 0.146	H atoms treated by a mixture of independent and constrained refinement
*S* = 1.09	*w* = 1/[σ^2^(*F*_o_^2^) + (0.0654*P*)^2^ + 1.6916*P*] where *P* = (*F*_o_^2^ + 2*F*_c_^2^)/3
5036 reflections	(Δ/σ)_max_ = 0.008
344 parameters	Δρ_max_ = 0.18 e Å^−^^3^
2 restraints	Δρ_min_ = −0.31 e Å^−^^3^

### Special details

**Table:**

Geometry. Bond distances, angles etc. have been calculated using the rounded fractional coordinates. All su's are estimated from the variances of the (full) variance-covariance matrix. The cell esds are taken into account in the estimation of distances, angles and torsion angles
Refinement. Refinement of *F*^2^ against ALL reflections. The weighted *R*-factor *wR* and goodness of fit *S* are based on *F*^2^, conventional *R*-factors *R* are based on *F*, with *F* set to zero for negative *F*^2^. The threshold expression of *F*^2^ > σ(*F*^2^) is used only for calculating *R*-factors(gt) *etc*. and is not relevant to the choice of reflections for refinement. *R*-factors based on *F*^2^ are statistically about twice as large as those based on *F*, and *R*- factors based on ALL data will be even larger.

### Fractional atomic coordinates and isotropic or equivalent isotropic displacement parameters (Å^2^)

**Table:**

	*x*	*y*	*z*	*U*_iso_*/*U*_eq_	Occ. (<1)
O1	0.15295 (6)	0.1181 (2)	0.38244 (6)	0.0831 (6)	
O2	0.20401 (5)	0.0099 (2)	0.31780 (6)	0.0765 (6)	
O3	0.12749 (6)	0.0181 (2)	0.05234 (7)	0.0902 (7)	
O4	0.06211 (5)	−0.14990 (17)	0.04359 (6)	0.0727 (5)	
O5	0.03119 (9)	0.5972 (2)	0.17358 (9)	0.1146 (9)	
O6	0.10758 (7)	0.50789 (18)	0.14631 (8)	0.0950 (7)	
N1	−0.03049 (6)	0.1524 (2)	0.13608 (7)	0.0614 (6)	
C1	0.26807 (8)	−0.0271 (3)	0.51329 (8)	0.0651 (7)	
C2	0.26107 (10)	0.0205 (4)	0.56879 (10)	0.0966 (10)	
C3	0.29518 (13)	−0.0298 (4)	0.61298 (11)	0.1089 (13)	
C4	0.33541 (12)	−0.1280 (4)	0.60133 (13)	0.0984 (11)	
C5	0.34198 (10)	−0.1753 (3)	0.54686 (13)	0.0945 (11)	
C6	0.30910 (8)	−0.1260 (3)	0.50304 (10)	0.0761 (8)	
C7	0.23098 (7)	0.0247 (3)	0.46755 (9)	0.0661 (7)	
C8	0.23225 (7)	−0.0054 (2)	0.41294 (8)	0.0610 (7)	
C9	0.19188 (7)	0.0489 (3)	0.37172 (8)	0.0603 (7)	
C10	0.17077 (7)	0.0613 (3)	0.27168 (8)	0.0617 (7)	
C11	0.19264 (8)	0.1538 (3)	0.23170 (9)	0.0793 (9)	
C12	0.16255 (8)	0.1999 (3)	0.18433 (9)	0.0749 (8)	
C13	0.11058 (6)	0.1577 (2)	0.17750 (7)	0.0510 (6)	
C14	0.09000 (7)	0.0638 (2)	0.21843 (7)	0.0540 (6)	
C15	0.12003 (7)	0.0140 (2)	0.26555 (8)	0.0597 (7)	
C16	0.07650 (7)	0.2137 (2)	0.12629 (7)	0.0533 (6)	
C17	0.03766 (7)	0.3350 (2)	0.14437 (7)	0.0555 (6)	
C18	−0.01294 (8)	0.2973 (2)	0.15185 (8)	0.0583 (7)	
C19	−0.00213 (7)	0.0484 (2)	0.10495 (7)	0.0536 (6)	
C20	0.04903 (7)	0.0777 (2)	0.09681 (7)	0.0510 (6)	
C21	0.08341 (8)	−0.0176 (3)	0.06266 (7)	0.0589 (7)	
C22	0.09348 (9)	−0.2500 (3)	0.00904 (10)	0.0840 (9)	
C23	0.06010 (12)	−0.3865 (3)	−0.00605 (12)	0.1018 (11)	
C24	−0.03430 (8)	−0.0877 (3)	0.08445 (9)	0.0702 (8)	
C25	−0.05506 (9)	0.3959 (3)	0.17583 (11)	0.0842 (9)	
C26	0.05650 (11)	0.4907 (3)	0.15686 (9)	0.0737 (9)	
C27A	0.1365 (2)	0.6471 (6)	0.1590 (2)	0.091 (2)	0.716 (5)
C28A	0.15865 (17)	0.6335 (5)	0.2190 (2)	0.1116 (19)	0.716 (5)
C28B	0.1719 (5)	0.6871 (14)	0.1717 (6)	0.1116 (19)	0.284 (5)
C27B	0.1144 (5)	0.6777 (12)	0.1772 (5)	0.072 (4)	0.284 (5)
O1W	−0.0134 (3)	0.8162 (4)	0.2322 (3)	0.143 (4)	0.500
H3	0.29060	0.00340	0.65040	0.1310*	
H4	0.35820	−0.16220	0.63080	0.1180*	
H2	0.23350	0.08660	0.57680	0.1160*	
H6	0.31440	−0.15950	0.46580	0.0910*	
H7	0.20330	0.08640	0.47890	0.0790*	
H5	0.36930	−0.24260	0.53910	0.1130*	
H11	0.22740	0.18550	0.23630	0.0950*	
H12	0.17760	0.26060	0.15640	0.0900*	
H14	0.05510	0.03300	0.21440	0.0650*	
H15	0.10570	−0.05090	0.29270	0.0720*	
H16	0.09960	0.26170	0.09870	0.0640*	
H22A	0.12500	−0.28220	0.03050	0.1010*	
H8	0.26000	−0.06390	0.39970	0.0730*	
H1N	−0.0639 (6)	0.136 (2)	0.1384 (9)	0.0740*	
H23B	0.02800	−0.35200	−0.02490	0.1530*	
H23C	0.05220	−0.44200	0.02830	0.1530*	
H24A	−0.07040	−0.07110	0.09310	0.1050*	
H24B	−0.02170	−0.18010	0.10340	0.1050*	
H24C	−0.03150	−0.09900	0.04370	0.1050*	
H25A	−0.08780	0.34060	0.17370	0.1260*	
H25B	−0.05840	0.49020	0.15400	0.1260*	
H25C	−0.04600	0.42040	0.21520	0.1260*	
H27A	0.11360	0.73670	0.15550	0.1090*	0.716 (5)
H27B	0.16460	0.65930	0.13240	0.1090*	0.716 (5)
H28A	0.13050	0.63070	0.24520	0.1680*	0.716 (5)
H28B	0.18080	0.72120	0.22770	0.1680*	0.716 (5)
H28C	0.17890	0.53980	0.22280	0.1680*	0.716 (5)
H22B	0.10370	−0.19640	−0.02540	0.1010*	
H23A	0.07850	−0.45360	−0.03130	0.1530*	
H27C	0.09580	0.75920	0.15620	0.0860*	0.284 (5)
H27D	0.10430	0.67670	0.21690	0.0860*	0.284 (5)
H28D	0.18840	0.60140	0.19160	0.1680*	0.284 (5)
H28E	0.18470	0.78280	0.18820	0.1680*	0.284 (5)
H28F	0.18010	0.68320	0.13180	0.1680*	0.284 (5)
H1W	0.01850	0.74420	0.22660	0.2150*	

### Atomic displacement parameters (Å^2^)

**Table:**

	*U*^11^	*U*^22^	*U*^33^	*U*^12^	*U*^13^	*U*^23^
O1	0.0591 (9)	0.1313 (14)	0.0581 (8)	0.0294 (9)	−0.0094 (7)	−0.0096 (8)
O2	0.0594 (8)	0.1200 (13)	0.0496 (8)	0.0294 (8)	−0.0052 (6)	0.0052 (8)
O3	0.0572 (9)	0.1244 (14)	0.0903 (11)	−0.0163 (9)	0.0193 (8)	−0.0238 (10)
O4	0.0681 (9)	0.0813 (10)	0.0693 (9)	−0.0021 (8)	0.0110 (7)	−0.0154 (8)
O5	0.1437 (17)	0.0679 (11)	0.1328 (17)	−0.0098 (12)	0.0121 (14)	−0.0171 (11)
O6	0.0896 (12)	0.0735 (10)	0.1188 (14)	−0.0290 (9)	−0.0366 (10)	0.0151 (9)
N1	0.0445 (8)	0.0724 (11)	0.0672 (10)	−0.0046 (8)	0.0017 (7)	−0.0069 (8)
C1	0.0518 (11)	0.0847 (14)	0.0578 (12)	−0.0103 (10)	−0.0103 (9)	0.0094 (10)
C2	0.0859 (17)	0.139 (2)	0.0634 (15)	0.0026 (16)	−0.0145 (12)	−0.0060 (15)
C3	0.114 (2)	0.151 (3)	0.0594 (15)	−0.025 (2)	−0.0245 (15)	0.0058 (16)
C4	0.0914 (19)	0.105 (2)	0.095 (2)	−0.0268 (16)	−0.0439 (16)	0.0313 (16)
C5	0.0811 (16)	0.0976 (19)	0.102 (2)	0.0023 (14)	−0.0309 (14)	0.0253 (16)
C6	0.0664 (13)	0.0879 (16)	0.0727 (14)	0.0054 (12)	−0.0143 (11)	0.0142 (12)
C7	0.0486 (10)	0.0906 (15)	0.0586 (12)	0.0048 (10)	−0.0059 (9)	0.0010 (10)
C8	0.0473 (10)	0.0794 (14)	0.0559 (11)	0.0081 (9)	−0.0033 (8)	0.0072 (10)
C9	0.0471 (10)	0.0817 (14)	0.0516 (11)	0.0034 (10)	−0.0039 (8)	0.0038 (10)
C10	0.0518 (11)	0.0883 (14)	0.0443 (10)	0.0166 (10)	−0.0059 (8)	0.0008 (10)
C11	0.0454 (11)	0.129 (2)	0.0627 (13)	−0.0076 (12)	−0.0089 (9)	0.0161 (13)
C12	0.0529 (11)	0.1111 (18)	0.0601 (12)	−0.0177 (11)	−0.0072 (9)	0.0241 (12)
C13	0.0466 (9)	0.0619 (11)	0.0440 (9)	−0.0036 (8)	−0.0032 (7)	0.0011 (8)
C14	0.0469 (9)	0.0667 (12)	0.0481 (10)	−0.0043 (8)	−0.0025 (8)	0.0009 (9)
C15	0.0597 (12)	0.0736 (13)	0.0459 (10)	0.0013 (9)	0.0017 (8)	0.0070 (9)
C16	0.0486 (10)	0.0658 (12)	0.0450 (9)	−0.0115 (9)	−0.0052 (7)	0.0077 (8)
C17	0.0606 (11)	0.0602 (11)	0.0444 (9)	−0.0046 (9)	−0.0136 (8)	0.0061 (8)
C18	0.0618 (12)	0.0621 (12)	0.0501 (10)	0.0050 (9)	−0.0097 (8)	0.0007 (9)
C19	0.0493 (10)	0.0658 (12)	0.0454 (9)	−0.0039 (9)	−0.0034 (8)	0.0000 (8)
C20	0.0476 (10)	0.0656 (12)	0.0393 (9)	−0.0069 (8)	−0.0048 (7)	0.0035 (8)
C21	0.0536 (11)	0.0802 (14)	0.0426 (10)	−0.0034 (10)	−0.0036 (8)	0.0012 (9)
C22	0.0838 (15)	0.1019 (18)	0.0666 (13)	0.0154 (14)	0.0085 (11)	−0.0132 (13)
C23	0.135 (2)	0.0873 (18)	0.0831 (17)	0.0083 (17)	0.0029 (16)	−0.0220 (14)
C24	0.0535 (11)	0.0794 (14)	0.0772 (14)	−0.0121 (10)	−0.0018 (10)	−0.0123 (11)
C25	0.0754 (15)	0.0865 (17)	0.0900 (16)	0.0186 (12)	−0.0045 (12)	−0.0134 (13)
C26	0.0996 (18)	0.0606 (14)	0.0589 (12)	−0.0084 (13)	−0.0206 (12)	0.0097 (10)
C27A	0.083 (4)	0.076 (3)	0.113 (4)	−0.016 (3)	0.000 (2)	−0.011 (3)
C28A	0.098 (3)	0.097 (3)	0.136 (4)	−0.003 (2)	−0.044 (3)	−0.029 (3)
C28B	0.098 (3)	0.097 (3)	0.136 (4)	−0.003 (2)	−0.044 (3)	−0.029 (3)
C27B	0.076 (7)	0.059 (6)	0.081 (7)	−0.015 (5)	0.003 (5)	−0.012 (5)
O1W	0.179 (8)	0.082 (2)	0.175 (8)	0.041 (3)	0.095 (6)	0.024 (3)

### Geometric parameters (Å, º)

**Table:**

O1—C9	1.193 (3)	C20—C21	1.459 (3)
O2—C9	1.348 (2)	C22—C23	1.486 (4)
O2—C10	1.413 (2)	C27A—C28A	1.491 (7)
O3—C21	1.198 (3)	C27B—C28B	1.479 (18)
O4—C21	1.332 (3)	C2—H2	0.9300
O4—C22	1.443 (3)	C3—H3	0.9300
O5—C26	1.196 (3)	C4—H4	0.9300
O6—C26	1.343 (3)	C5—H5	0.9300
O6—C27A	1.433 (5)	C6—H6	0.9300
O6—C27B	1.637 (11)	C7—H7	0.9300
O1W—H1W	1.0400	C8—H8	0.9300
O1W—H1W^i^	1.1500	C11—H11	0.9300
N1—C19	1.376 (2)	C12—H12	0.9300
N1—C18	1.372 (2)	C14—H14	0.9300
N1—H1N	0.867 (15)	C15—H15	0.9300
C1—C2	1.374 (3)	C16—H16	0.9800
C1—C7	1.466 (3)	C22—H22B	0.9700
C1—C6	1.377 (3)	C22—H22A	0.9700
C2—C3	1.390 (4)	C23—H23A	0.9600
C3—C4	1.365 (5)	C23—H23B	0.9600
C4—C5	1.349 (4)	C23—H23C	0.9600
C5—C6	1.363 (4)	C24—H24A	0.9600
C7—C8	1.300 (3)	C24—H24C	0.9600
C8—C9	1.457 (3)	C24—H24B	0.9600
C10—C15	1.358 (3)	C25—H25B	0.9600
C10—C11	1.362 (3)	C25—H25C	0.9600
C11—C12	1.377 (3)	C25—H25A	0.9600
C12—C13	1.377 (3)	C27A—H27A	0.9700
C13—C16	1.526 (2)	C27A—H27B	0.9700
C13—C14	1.370 (2)	C27B—H27C	0.9700
C14—C15	1.382 (2)	C27B—H27D	0.9700
C16—C17	1.511 (2)	C28A—H28A	0.9600
C16—C20	1.516 (2)	C28A—H28B	0.9600
C17—C26	1.451 (3)	C28A—H28C	0.9600
C17—C18	1.348 (3)	C28B—H28F	0.9600
C18—C25	1.494 (3)	C28B—H28D	0.9600
C19—C24	1.498 (3)	C28B—H28E	0.9600
C19—C20	1.349 (3)		
			
C9—O2—C10	118.53 (16)	C5—C6—H6	120.00
C21—O4—C22	118.05 (16)	C1—C6—H6	120.00
C26—O6—C27A	123.3 (3)	C8—C7—H7	116.00
C26—O6—C27B	96.1 (5)	C1—C7—H7	116.00
H1W—O1W—H1W^i^	84.00	C7—C8—H8	119.00
C18—N1—C19	124.16 (16)	C9—C8—H8	119.00
C18—N1—H1N	116.3 (12)	C12—C11—H11	120.00
C19—N1—H1N	117.7 (12)	C10—C11—H11	120.00
C2—C1—C6	118.4 (2)	C11—C12—H12	119.00
C6—C1—C7	122.35 (19)	C13—C12—H12	119.00
C2—C1—C7	119.3 (2)	C15—C14—H14	119.00
C1—C2—C3	120.2 (3)	C13—C14—H14	119.00
C2—C3—C4	119.9 (3)	C10—C15—H15	120.00
C3—C4—C5	119.7 (3)	C14—C15—H15	120.00
C4—C5—C6	121.0 (3)	C13—C16—H16	108.00
C1—C6—C5	120.8 (2)	C17—C16—H16	108.00
C1—C7—C8	127.62 (19)	C20—C16—H16	108.00
C7—C8—C9	122.47 (18)	O4—C22—H22B	110.00
O2—C9—C8	110.62 (16)	C23—C22—H22A	111.00
O1—C9—C8	126.50 (18)	C23—C22—H22B	111.00
O1—C9—O2	122.88 (17)	H22A—C22—H22B	109.00
O2—C10—C11	117.00 (17)	O4—C22—H22A	110.00
O2—C10—C15	121.74 (18)	C22—C23—H23A	109.00
C11—C10—C15	121.16 (18)	C22—C23—H23B	109.00
C10—C11—C12	119.12 (19)	H23A—C23—H23B	110.00
C11—C12—C13	121.3 (2)	H23A—C23—H23C	109.00
C12—C13—C16	121.50 (16)	H23B—C23—H23C	109.00
C14—C13—C16	120.58 (15)	C22—C23—H23C	110.00
C12—C13—C14	117.92 (17)	C19—C24—H24B	109.00
C13—C14—C15	121.38 (16)	C19—C24—H24C	109.00
C10—C15—C14	119.08 (17)	C19—C24—H24A	109.00
C13—C16—C20	110.39 (14)	H24A—C24—H24C	109.00
C17—C16—C20	111.40 (15)	H24B—C24—H24C	110.00
C13—C16—C17	111.11 (13)	H24A—C24—H24B	109.00
C18—C17—C26	120.53 (18)	C18—C25—H25B	109.00
C16—C17—C26	118.71 (17)	C18—C25—H25C	109.00
C16—C17—C18	120.67 (16)	C18—C25—H25A	110.00
N1—C18—C17	119.24 (17)	H25A—C25—H25C	110.00
N1—C18—C25	112.71 (18)	H25B—C25—H25C	109.00
C17—C18—C25	128.05 (17)	H25A—C25—H25B	109.00
C20—C19—C24	128.45 (17)	O6—C27A—H27B	110.00
N1—C19—C24	112.67 (16)	C28A—C27A—H27A	110.00
N1—C19—C20	118.88 (16)	C28A—C27A—H27B	110.00
C16—C20—C19	120.87 (15)	H27A—C27A—H27B	108.00
C16—C20—C21	113.91 (16)	O6—C27A—H27A	110.00
C19—C20—C21	125.15 (17)	O6—C27B—H27D	113.00
O3—C21—O4	121.8 (2)	C28B—C27B—H27C	113.00
O3—C21—C20	123.6 (2)	H27C—C27B—H27D	110.00
O4—C21—C20	114.70 (17)	O6—C27B—H27C	113.00
O4—C22—C23	106.29 (19)	C28B—C27B—H27D	113.00
O5—C26—C17	126.6 (3)	H28A—C28A—H28C	109.00
O5—C26—O6	121.0 (2)	H28B—C28A—H28C	109.00
O6—C26—C17	112.4 (2)	C27A—C28A—H28A	109.00
O6—C27A—C28A	107.5 (4)	C27A—C28A—H28B	110.00
O6—C27B—C28B	95.7 (8)	H28A—C28A—H28B	109.00
C3—C2—H2	120.00	C27A—C28A—H28C	110.00
C1—C2—H2	120.00	C27B—C28B—H28E	110.00
C2—C3—H3	120.00	C27B—C28B—H28F	110.00
C4—C3—H3	120.00	C27B—C28B—H28D	109.00
C5—C4—H4	120.00	H28D—C28B—H28F	109.00
C3—C4—H4	120.00	H28E—C28B—H28F	109.00
C4—C5—H5	120.00	H28D—C28B—H28E	109.00
C6—C5—H5	119.00		
			
C10—O2—C9—O1	3.9 (3)	C11—C12—C13—C16	177.6 (2)
C10—O2—C9—C8	−176.27 (18)	C12—C13—C14—C15	0.7 (3)
C9—O2—C10—C11	119.8 (2)	C16—C13—C14—C15	−178.96 (16)
C9—O2—C10—C15	−63.7 (3)	C12—C13—C16—C17	−107.2 (2)
C22—O4—C21—O3	−0.8 (3)	C12—C13—C16—C20	128.71 (19)
C22—O4—C21—C20	179.46 (16)	C14—C13—C16—C17	72.5 (2)
C21—O4—C22—C23	−179.81 (18)	C14—C13—C16—C20	−51.6 (2)
C27A—O6—C26—O5	6.0 (4)	C13—C14—C15—C10	1.0 (3)
C27A—O6—C26—C17	−175.4 (3)	C13—C16—C17—C18	−101.32 (19)
C26—O6—C27A—C28A	89.2 (4)	C13—C16—C17—C26	75.0 (2)
C19—N1—C18—C17	−10.2 (3)	C20—C16—C17—C18	22.2 (2)
C19—N1—C18—C25	169.52 (18)	C20—C16—C17—C26	−161.42 (16)
C18—N1—C19—C20	11.1 (3)	C13—C16—C20—C19	102.65 (18)
C18—N1—C19—C24	−169.25 (17)	C13—C16—C20—C21	−74.51 (18)
C6—C1—C2—C3	0.6 (4)	C17—C16—C20—C19	−21.3 (2)
C7—C1—C2—C3	178.7 (3)	C17—C16—C20—C21	161.55 (15)
C2—C1—C6—C5	−0.1 (4)	C16—C17—C18—N1	−8.0 (3)
C7—C1—C6—C5	−178.1 (2)	C16—C17—C18—C25	172.33 (19)
C2—C1—C7—C8	178.2 (3)	C26—C17—C18—N1	175.67 (17)
C6—C1—C7—C8	−3.8 (4)	C26—C17—C18—C25	−4.0 (3)
C1—C2—C3—C4	−0.7 (5)	C16—C17—C26—O5	−177.1 (2)
C2—C3—C4—C5	0.3 (5)	C16—C17—C26—O6	4.4 (3)
C3—C4—C5—C6	0.3 (5)	C18—C17—C26—O5	−0.7 (3)
C4—C5—C6—C1	−0.4 (4)	C18—C17—C26—O6	−179.25 (18)
C1—C7—C8—C9	177.9 (2)	N1—C19—C20—C16	6.2 (2)
C7—C8—C9—O1	−3.8 (4)	N1—C19—C20—C21	−177.00 (17)
C7—C8—C9—O2	176.4 (2)	C24—C19—C20—C16	−173.42 (17)
O2—C10—C11—C12	176.5 (2)	C24—C19—C20—C21	3.4 (3)
C15—C10—C11—C12	0.1 (4)	C16—C20—C21—O3	−9.3 (3)
O2—C10—C15—C14	−177.68 (18)	C16—C20—C21—O4	170.37 (15)
C11—C10—C15—C14	−1.4 (3)	C19—C20—C21—O3	173.65 (19)
C10—C11—C12—C13	1.8 (4)	C19—C20—C21—O4	−6.7 (3)
C11—C12—C13—C14	−2.1 (3)		

Symmetry code: (i) −*x*, *y*, −*z*+1/2.

### Hydrogen-bond geometry (Å, º)

**Table:**

*D*—H···*A*	*D*—H	H···*A*	*D*···*A*	*D*—H···*A*
N1—H1*N*···O1^i^	0.87 (2)	2.31 (2)	3.148 (2)	163 (2)
O1*W*—H1*W*···O5	1.04	1.81	2.615 (6)	132
C14—H14···O1*W*^ii^	0.93	2.50	3.140 (6)	126

Symmetry codes: (i) −*x*, *y*, −*z*+1/2; (ii) −*x*, *y*−1, −*z*+1/2.
